# Choosing the Right Therapy for Patients with Relapsed/Refractory Multiple Myeloma (RRMM) in Consideration of Patient-, Disease- and Treatment-Related Factors

**DOI:** 10.3390/cancers13174320

**Published:** 2021-08-26

**Authors:** Laura Gengenbach, Giulia Graziani, Heike Reinhardt, Amelie Rösner, Magdalena Braun, Mandy-Deborah Möller, Christine Greil, Ralph Wäsch, Monika Engelhardt

**Affiliations:** University Medical Center Freiburg, Department of Hematology, Oncology & Stem Cell Transplantation, Faculty of Medicine, University of Freiburg, 79106 Freiburg, Germany; laura.gengenbach@uniklinik-freiburg.de (L.G.); giulia.graziani@uniklinik-freiburg.de (G.G.); heike.reinhardt@uniklinik-freiburg.de (H.R.); amelie.roesner@uniklinik-freiburg.de (A.R.); magdalena.braun@uniklinik-freiburg.de (M.B.); mandy-deborah.moeller@uniklinik-freiburg.de (M.-D.M.); christine.greil@uniklinik-freiburg.de (C.G.); ralph.waesch@uniklinik-freiburg.de (R.W.)

**Keywords:** relapsed/refractory multiple myeloma (RRMM), novel agents, immunotherapy, frailty, geriatric assessment (GA), revised myeloma comorbidity Index (R-MCI)

## Abstract

**Simple Summary:**

During the course of their disease, almost all multiple myeloma patients experience one or more relapses. Treatment options for relapsed/refractory multiple myeloma (RRMM) have largely increased in the last decades. The choice of when and how to treat in the relapsed/refractory setting can therefore be challenging. Since multiple myeloma (MM) typically affects elderly people, it is of importance to include specific, frailty-related comorbidities in the choice of treatment. The aim of this review was to present an update on treatment options for patients with RRMM, under consideration of today’s available literature, current guidelines and patient-, disease- and treatment-related factors. We focused on geriatric assessments (GA) and frailty scores such as the revised myeloma comorbidity index (R-MCI), international myeloma working group (IMWG-) frailty index and others as these allow obtaining a more accurate picture of the individual patient constitution than the numerical age alone.

**Abstract:**

Treatment of relapsed/refractory multiple myeloma (RRMM) is more complex today due to the availability of novel therapeutic options, mostly applied as combination regimens. immunotherapy options have especially increased substantially, likewise the understanding that patient-, disease- and treatment-related factors should be considered at all stages of the disease. RRMM is based on definitions of the international myeloma working group (IMWG) and includes biochemical progression, such as paraprotein increase, or symptomatic relapse with CRAB criteria (hypercalcemia, renal impairment, anemia, bone lesions). When choosing RRMM-treatment, the biochemical markers for progression and severity of the disease, dynamic of disease relapse, type and number of prior therapy lines, including toxicity and underlying health status, need to be considered, and shared decision making should be pursued. Objectively characterizing health status via geriatric assessment (GA) at each multiple myeloma (MM) treatment decision point has been shown to be a better estimate than via age and comorbidities alone. The well-established national comprehensive cancer network, IMWG, European myeloma network and other national treatment algorithms consider these issues. Ideally, GA-based clinical trials should be supported in the future to choose wisely and efficaciously from available intervention and treatment options in often-older MM adults in order to further improve morbidity and mortality.

## 1. Introduction

MM is the second most common hematological malignancy and is characterized by clonal proliferation of plasma cells in the bone marrow or less frequently at extramedullary sites producing monoclonal immunoglobulins [1]. This can lead to various symptoms such as hypercalcemia, renal impairment, anemia/pancytopenia, osteolysis—including bone pain, hyperviscosity and immunoparesis with susceptibility to infections and/or polyneuropathy.

Symptomatic, therapy-requiring MM is defined by the CRAB and SLIM-CRAB criteria [2]. SLIM criteria include biomarkers of malignancy indicating a higher risk of progression from smoldering multiple myeloma (SMM) to symptomatic myeloma, i.e., reaching CRAB criteria of >80% within two years. SLIM-CRAB criteria include an infiltration rate of bone marrow plasma cells ≥60%, an involved/uninvolved serum free light chain (SFLC) ratio of ≥100 or more than one focal lesion of ≥5 mm present on magnetic resonance imaging (MRI).

According to the guidelines of the IMWG, therapy is indicated and should be discussed in symptomatic MM with ≥1 CRAB criteria. The updated IMWG-guidelines recommend early initiation of therapy to prevent end-organ damage in patients who are at a high risk of progression to symptomatic disease, defined by the presence of ≥1 biomarker of malignancy (SLIM-CRAB criteria) [2].

**Figure 1:**
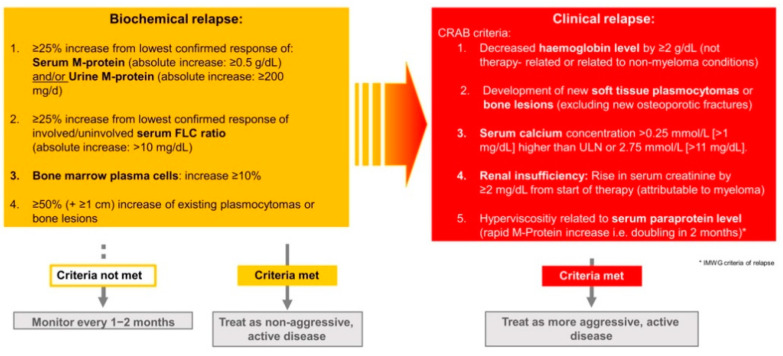
Definition of relapse and indication for treatment. Since MM is an incurable disease, almost all patients experience at least one or more relapses over the course of their disease. It is of great importance to define the right time point to restart or change therapy. The CRAB criteria as well as the IMWG criteria, representing defined biochemical markers of relevance for disease outcome, indicate the need for antimyeloma treatment. If a myeloma patient does not present with typical symptoms or if an increase in biochemical markers is not significant enough, therapy is not imminent; instead, close monitoring of the patient and the decisive diagnostics every 1–2 months should follow. Abbreviations: CRAB criteria = hypercalcemia, renal failure, anemia, bone lesions, FLC = free light chain (monoclonal immunoglobulin light chain), IMWG = international myeloma working group, M-protein = abnormal antibody/monoclonal proliferation of plasma cells, ULN = upper limit of normal.

Not only treatment initiation, but relapse treatment in MM is a highly discussed topic today, because nearly all myeloma patients experience at least one or multiple relapses during the course of their disease. In case of symptomatic relapse, defined by aggravated or new end-organ damage, there is a clear indication to restart or switch antimyeloma treatment. Besides clinical relapse criteria, there are established markers of biochemical progression (Figure 1), which need to be considered regarding the optimal time point of re-initiation or change of treatment [3,4].

In case of confirmed RRMM, the choice of when and how to treat can be challenging due to different patient-, disease- and treatment-related factors. Preexisting and myeloma-related comorbidities can make choosing the most effective antimyeloma treatment, expected toxicities and quality of life (QoL) preservation demanding. Moreover, the treatment of RRMM has become more diverse, because novel insights, better understanding of the disease biology and clinical trials have led to the development and approval of various new antimyeloma drugs and combinations [5]. These may include first-, second- and third-generation immunomodulatory drugs (IMiDs), or cereblon-modifying agents, proteasome inhibitors (PIs) and targeted agents such as monoclonal antibodies (mAbs), antibody drug conjugates, bispecific T-cell engagers (BiTEs), chimeric antigen receptor T-cells (CAR-T-cells), belantamab, selinexor, venetoclax and various others.

Because of these complex therapeutic aspects, ideally requiring the consideration of multiple factors, interdisciplinary conferences and tumor boards are essential in the process of decision-making today [6].

Myeloma typically affects elderly patients with a median age of 69 years, and survival may often be shorter in older patients [7]. The short survival is not only related to the underlying MM, as it is not considered more aggressive with aging. As older patients often present with a more advanced disease stage, comorbidities and frailty, this may increase the quantity of therapy-related toxicities and may allow less therapy lines to be exerted. Therefore, this may lead to fewer effective therapies being performed in elderly and/or frail MM patients [8,9].

Nevertheless, the numerical age alone has proven insufficient for choosing the best therapeutic option, since elderly RRMM patients are heterogeneous in their performance status, endurance of and willingness to undergo antimyeloma therapy. This patient heterogeneity underlines the importance of using subjective and objective tools to optimize and individualize antimyeloma treatment, with the aim to avoid undertreatment of fit and overtreatment of frail patients. GA and frailty scores such as the revised myeloma comorbidity index (R-MCI), the IMWG frailty-index and others can help to overcome this therapeutic dilemma [10,11,12,13].

This review provides an overview of the diagnosis of RRMM, when and how to treat and how to find the best individual treatment option for each patient.

## 2. Diagnosis of RRMM

Disease progression in myeloma is monitored by the measurement of M-protein in blood and urine as well as serum protein electrophoresis, serum and urine immunofixation, SFLC-assay, additional laboratory parameters, imaging and bone marrow aspirate/biopsy. Serum beta-2-microglobuline and serum lactate dehydrogenase (LDH) are useful biomarkers due to their diagnostic and prognostic value [14]. In order to assess clinical relapse, serum calcium levels, serum creatinine/estimated glomerular filtration rate (eGFR) and differential blood count are acquired [15].

A bone marrow aspirate or biopsy should be conducted, since this allows determining the degree of bone marrow infiltration by aberrant plasma cells and persistence or gain of cytogenetic abnormalities via fluorescence in situ hybridization (FISH). An additional bone marrow biopsy can be valuable to perform, due to some patients with packed marrow making it difficult to acquire BM aspirates. This may ensure that at least one valid BM assessment is available and BM infiltration can be evaluated, if no aspirate is being obtained or if both show larger discrepancies [16,17,18]. In patients with clear relapse due to an increase in M-protein, detected by serum protein electrophoresis and/or SFLCs and ratio, a bone marrow puncture might be omitted as an individual patient and physician decision [15].

In case of relapse, a repeated assessment of individual patients’ risks is recommended in order to account for clonal evolution and to obtain a currently valid evaluation. The revised international staging system (R-ISS) should be reassessed, which includes the ISS, cytogenetics by FISH and LDH. In addition, other risk factors such as extramedullary disease, circulating plasma cells, comorbidities such as renal or other organ impairment and frailty by GA should be examined [19].

Precise disease monitoring involves minimal residual disease (MRD)-assessment on a regular basis at least within clinical trials, since studies have shown that MRD-negativity is associated with improved response, progression-free (PFS) and overall survival (OS) [20,21]. Methods of MRD-assessment in the bone marrow include multiparametric flow cytometry (MFC) and molecular methods such as next-generation sequencing. The required sensitivity is 1 in 10^−4^–10^−6^, ideally 1/10^−5^ nucleated cells [22,23].

The detection of extramedullary disease for MRD assessment can be performed via fluorodeoxyglucose positron emission tomography (FDG-PET) [3]. Complete response ((CR) as negative urine and serum immunofixation, <5% plasma cells in the bone marrow and disappearance of any soft tissue plasmocytomas) is defined as MRD-negativity by the IMWG [3] either by next-generation flow cytometry or next-generation sequencing in absence of phenotypically aberrant or clonal plasma cells with a minimum sensitivity of 1 in 10^−^⁵ nucleated cells or higher [3].

In RRMM, a whole-body low-dose computer tomography (WB-CT) is the preferred technique of imaging to assess new or progressive osteolytic bone lesions. MRI can be obtained as a more sensitive measurement regarding diffuse bone marrow infiltration, extramedullary manifestations and disease burden. Additionally, it is useful to detect focal bone marrow lesions, which can occur before osteolytic lesions and are visible via WB-CT. Conventional X-rays are imprecise and detect osteolytic or progressive osteodestructive lesions much later; therefore, WB-CT and MRI are ‘gold standards’ today [24,25]. The advantage of WB-CT compared with MRI is that it provides the possibility to assess the skeletal stability of osteolytic lesions. In case of suspected new or progressive known extramedullary disease, these imaging techniques can be combined with FDG-PET, which offers a high sensitivity for both medullary and extramedullary myeloma manifestations [26,27].

In patients with known extramedullary disease or soft tissue plasmocytomas upon initiation of treatment, imaging—preferably via FDG-PET or MRI—is useful as a key diagnostic procedure [28,29]. Patients presenting with pain, neurological or other symptoms indicating the occurrence of, e.g., spinal cord compression by extramedullary disease or fractured osteolytic bone lesions need to receive immediate imaging. Imaging remains valuable, even if diagnosis of RRMM via peripheral blood paraprotein increase, bone marrow and future MRD detection is made, because, in combination with thorough clinical examination of the patient, it allows to detect extramedullary disease [16,30].

Refractory myeloma is defined by non-responding disease under therapy, or prompt progression within 60 days after discontinued treatment [31].

## 3. Indications to Initiate Therapy

Recognizing the right time to start or change antimyeloma treatment in case of relapse is challenging and important at the same time. In case of clinical relapse, defined by new or aggravated CRAB criteria, end-organ damage such as hypercalcemia >11 mg/dL, myeloma-related rise in serum creatinine by ≥2 mg/dL from the start of therapy, myeloma-related decrease in hemoglobin level of ≥2 g/dL, new bone lesions, plasmocytomas or new hyperviscosity of the blood [3], treatment should be immediately initiated (Figure 1) [3,19]. Especially in case of rapid onset of symptoms, new extramedullary disease, high-risk cytogenetic abnormalities, doubling of the M-protein, elevated LDH, a high peripheral plasma cell count and high plasma cell proliferation index, RRMM should be treated as an aggressive disease [32].

If criteria of clinical relapse are not met, biochemical markers of disease progression need to be excluded. Biochemical relapse is defined by the IMWG as an increase in serum (absolute increase must be ≥0.5 g/dL) or urine M-protein (absolute increase must be ≥200 mg/24 h) ≥25% from the lowest response value, increased SFLC ratio ≥25% (absolute increase must be >10 mg/dL) from the lowest response value, increase of ≥10% of bone marrow plasma cells or increase in size of pre-existing bone lesions or plasmocytomas (Figure 1) [3]. Patients with confirmed biochemical relapse should receive therapy, especially in case of early, aggressive relapse, rapid increase in myeloma parameters and high-risk cytogenetics [19,33,34]. If the criteria of biochemical or clinical relapse are not met or in case of an asymptomatic, very slow increase in biochemical markers (evolving MM), close monitoring of myeloma parameters at least every 2–3 months is recommended [19,23].

## 4. Choosing the Right Therapy

**Figure 2:**
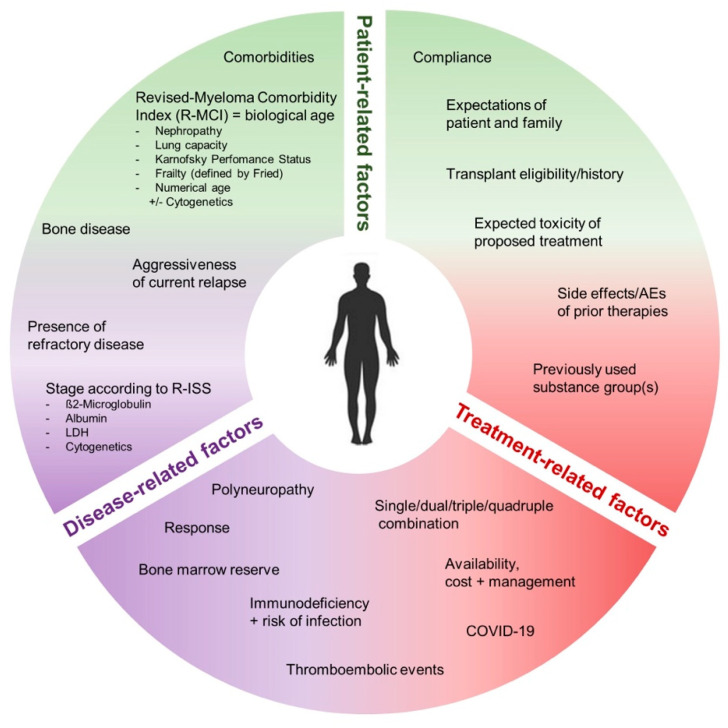
Relevant parameters for treatment selection in RRMM patients. In case of relapse, the choice of treatment should be adapted to patient-, disease- and treatment-related factors to define the best individual therapy for each patient. Over the course of the disease, an increase in myeloma-associated comorbidities, treatment toxicities and/or the natural process of aging make choosing the right treatment more difficult. Additionally, tolerability of previous treatments, the patient’s and family’s expectations, availability and management need to be considered. Abbreviations: AE = adverse events, COVID-19 = Coronavirus disease 2019, LDH = lactate dehydrogenase, R-ISS = revised international staging system, R-MCI = revised myeloma comorbidity index.

If relapse occurs, the choice of treatment should be adapted to disease-, treatment- and patient-related factors to find the optimal individual therapy for each patient (Figure 2).

Disease-related factors include the current stage according to R-ISS, cytogenetics, any end-organ damage, bone marrow reserve, immune status and depth and duration of the previously achieved remission. In addition, the aggressiveness and dynamics of relapse should be considered.

Moreover, treatment-related factors should be included: potential side effects and expected toxicities should be taken into account as well as the availability, costs and logistical differences of therapy administration (e.g., oral vs. intravenous route). The choice of therapy also strongly depends on the tolerability as well as depth and duration of remission induced by previously applied antimyeloma agents.

COVID-19-induced challenges for RRMM patients and possible consequences regarding the continuation of treatment have previously been described by IMWG, EMN and other MM/cancer experts [35,36,37]. General recommendations include the adherence to the prevailing preventive measures, such as social distancing, wearing a facemask and execution of thorough personal hygiene and broad vaccination against SARS-CoV-2 of patients, relatives and treating personnel. In specific clinical scenarios, remote consultations via telemedicine and—if possible—oralization and subcutaneous medication have also been suggested as valuable measures. In case of a low risk for infection with SARS-CoV-2 due to regional and personal factors, with vaccination or negative test results, antimyeloma treatment should be continued. Thus, successfully performed antimyeloma therapy should not be unnecessarily changed or deferred; it should rather be continued [37]. This includes supportives, such as bisphosphonates/denosumab, intravenous immunoglobulins and others. Vaccination against SARS-CoV-2 is strongly recommended for cancer and myeloma patients, ideally before the start of a systemic therapy [38,39,40], with more literature broadly being acquired on risks, SARS-CoV-2-management, vaccination strategies and ideally performed antimyeloma treatment. The interested readers are therefore referred to these articles, since this prevailing topic is beyond the scope of this review.

Additionally, it is relevant to incorporate patient-related factors into clinical decisions (Figure 2). Historically, numerical age and Karnofsky performance status (KPS) or eastern cooperative oncology group (ECOG) performance status were relevant parameters to define transplant eligibility or suitability for specific therapeutics, such as multi-agent vs. doublet or monotherapy regimens. Nevertheless, in heterogeneous RRMM patients and with increased diversity of effective and well-tolerated antimyeloma drugs [5], biological age and actual fitness, assessed via myeloma-specific comorbidity indices, such as the R-MCI or the IMWG frailty-index, become important as they allow a more objective patient assessment [10,11,13,41,42,43]. Originally, these comorbidity indices were developed for newly diagnosed MM (NDMM) patients. Nevertheless, in RRMM, any fitness assessment is even more important, because numerous relapse regimens exist and their selection is relevant to perform wisely, at best knowing the exact therapy endurance of an MM patient beforehand. Therefore, it is important to assess frailty and comorbidities over the disease course in MM tumor boards regularly and if new treatment decisions are being undertaken [6,44,45,46,47,48]. The assessment of changing performance status over the disease course was described by us in a reference AL-amyloidosis patient [46], in allogeneic stem cell transplantation-receiving MM patients [47], likewise in ASCT-receiving MM patients [48] and with use of quality of life (QoL) tests in various relapse phases [49]. All of these manuscripts verify that functional performance may change over time and is relevant for treatment decisions. QoL tests may deteriorate with each line of relapse treatment, making correct treatment decisions even more relevant [49]. Moreover, in our latest manuscript on the R-MCI [50], we had performed a detailed functional and risk score assessment at initial diagnosis and repeated it during follow-up (6–12 months after the initial assessment) to determine possible changes and capture differences in vulnerability and frailty in the course of treatment. Indeed, we found that functional tests and scores do improve over time, which was related to the obtained MM response and age. In fact, those patients achieving a partial remission or better (≥PR) and who were younger (<70 years of age) showed larger improvement than those with lesser response and the age of ≥70 years [50].

Regarding the expected toxicity and experienced side effects under prior therapies, it is important to include the patient’s expectations and wishes in the context of shared decision making and to take concomitant conditions into account as well. Since myeloma typically affects elderly patients, GA displays comorbidities and the level of frailty, thus helping to guide the decision making process with regard to each patient’s vulnerability [11,42,44,51,52,53]. Myeloma-associated comorbidities, such as skeletal lesions, fatigue and susceptibility to infections, can amplify factors of biological aging and frailty. Additionally, frail patients are at higher risk for treatment-related toxicities and adverse events (AEs) which can lead to treatment discontinuation and a higher mortality rate [54,55]. The identification of frail patients at risk therefore plays a crucial role in the process of defining suitable antimyeloma therapies. Ideally, GA serves as a reliable, objective and easy-to-use tool to evaluate a patient’s individual fitness status [11,13,42,51]. With this aim, the validated and prospectively assessed R-MCI was developed. It includes 5 multivariately determined weighted parameters: renal function (eGFR), pulmonary function measured by forced expiratory pressure in one second (FEV1) or substitutes, KPS, numerical age and frailty (according to Fried [56]). If available, cytogenetics can be included. Since it had repeatedly been requested by reviewers and myeloma experts to define “lung function” in the form of a more objective measure than via GOLD criteria, smoking status or dyspnea upon exertion alone, FEV1 was used for this and included as part of the R-MCI. Since lung function/FEV1 assessment is used in the diagnostic workup at primary to tertiary centers, likewise, i.e., before intensive treatment such as triplets, quadruplets or ASCT, FEV1-results are often available. If unavailable, smoking status (with cessation being mandatory before ASCT/intensive treatment), no advanced GOLD criteria and no advanced dyspnea without exertion have been used as substitutes in prior analyses [43,57,58,59]. If frailty definition according to Fried is unavailable, valuable substitutes were a KPS ≤ 70%, >10 s in the Timed up and Go Test (TUG), less than four points at the instrumental activities of daily living (IADL) scale and a subjectively impaired fitness grade rated by patients themselves (https://www.myelomacomorbidityindex.org/en_calc.html, Accessed 25 August 2021) [11,42,43,44]. Moreover, defined fitness tests and various comorbidity indices have been compared before and after at least 6 months of antimyeloma therapy in a large functional assessment. This prospective analysis defined which functional tests are of value to use in MM patients, and the precise group differences for fit, intermediate-fit and frail patients in OS and PFS were identified for the R-MCI, activity of daily living (ADL), the mini-mental state examination (MMSE), and quality-of-life (QoL) 12-item short form health survey physical composite scale (SF-12 PCS) [50].

The R-MCI categorizes patients into three different groups of “fit” (R-MCI score: 0–3), “intermediate-fit” (R-MCI score: 4–6) and “frail” (R-MCI score: 7–9) patients with significantly different PFS and OS (PFS: 4.1, 1.9, 0.9 years; OS: 10.1, 4.4, 1.2 years, respectively) [42]. The primary objective of such an assessment is to avoid over-treating of frail and under-treating fit elderly patients via individualized and adapted treatment strategies [10,51,60,61]. More objective insights of patients’ status via myeloma-specific GA can help to balance the often thin line between the best achievable treatment response, expected toxicities and health-related QoL [36]. Since elderly and frail patients are at risk of showing both severe AEs (SAEs) and treatment-related mortality [54,55], doses of antimyeloma agents should be adjusted to impaired organ functions such as renal, cardiac or liver function. Thus, the level of fitness vs. frailty needs to be deciphered, which, for the latter, may induce increased toxicities, treatment pauses or discontinuation, associated with shorter PFS and higher mortality [10,13,50,62,63,64].

**Figure 3:**
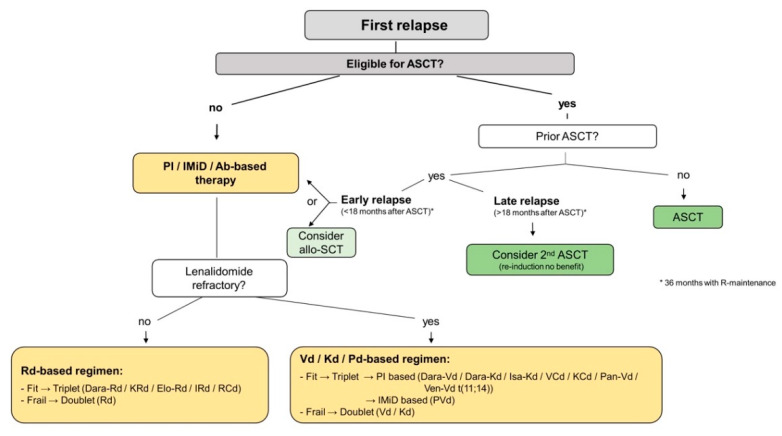
Treatment algorithm for RMM—1st relapse. Treatment algorithms for patients at first relapse aid in the process of decision-making and present numerous therapeutic options to be discussed with the patient. Relevant parameters for the use of a subsequent line of therapy are the duration of response to the last treatment, the eligibility for auto- or allo-SCT, the use of novel agents in first line therapy and its tolerability and achieved time of remission. Abbreviations: Ab = Antibody, allo-SCT = allogeneic stem cell transplantation, ASCT = autologous stem cell transplantation, Dara-Kd = daratumumab + carfilzomib + dexamethasone, Dara-Rd = daratumumab + lenalidomide + dexamethasone, Dara-Vd= daratumumab + bortezomib + dexamethasone, Elo-Rd = elotuzumab + lenalidomide + dexamethasone, IMiD = immunomodulatory drug, IRd = ixazomib + lenalidomide + dexamethasone, Isa-Kd = isatuximab + carfilzomib + dexamethasone, KCd = carfilzomib + bortezomib + dexamethasone, Kd = carfilzomib + dexamethasone, KRd = carfilzomib + lenalidomide + dexamethasone, Pan-Vd = panobinostat + bortezomib + dexamethasone, Pd = pomalidomide + dexamethasone, PI = proteasome inhibitor, PVd = pomalidomide + daratumumab + dexamethasone, R-maintenance = lenalidomide maintenance, RCd = lenalidomide + cyclophosphamide + dexamethasone, Rd = lenalidomide + dexamethasone, RRMM = relapsed refractory multiple myeloma, VCd = bortezomib + cyclophosphamide + dexamethasone, Vd = bortezomib + dexamethasone, Ven-Vd = venetoclax + bortezomib + dexamethasone.

**Figure 4:**
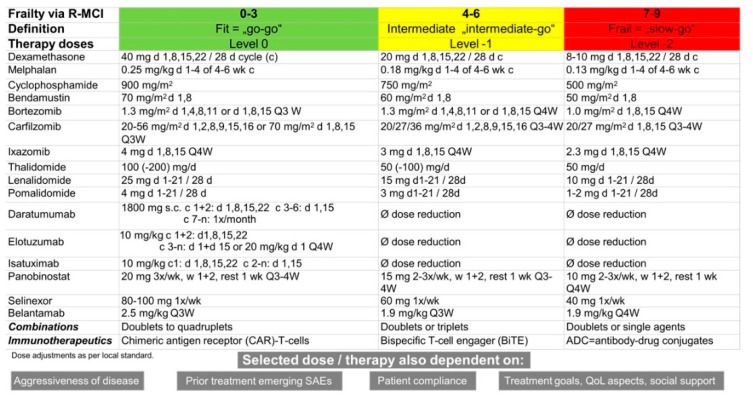
Dose adjustments regarding anti-MM therapy agents according to R-MCI. MM typically affects the elderly, this cohort of patients itself being very heterogeneous. Geriatric assessments such as the R-MCI or IMWG frailty index do not only show overt comorbidities but also display the level of frailty, which helps guide the process of decision making regarding each patient’s vulnerability. More precisely, the R-MCI is a validated and prospectively assessed comorbidity index, which groups patients into fit, intermediate and frail resulting in different PFS and OS for these patients. Consequently, treatment regimens and dosing of substances needs to be adapted according to the grade of frailty by R-MCI to prevent the overtreatment of frail patients and the undertreatment of fit patients. Abbreviations: c = cycle, d = day, Q3W = every 3 weeks, QoL = quality of life, R-MCI = revised myeloma comorbidity index, SAEs = serious adverse events, wk = week.

Monotherapies or doublets may also be considered for frail patients, whilst fit or intermediate-fit patients may receive triplets or quadruplets (Figure 3 and Figure 4) [19,30,65,66].

The chosen regimen and doses of antimyeloma agents should also depend on individualized treatment goals, which balance efficacy and expected toxicities, including patients’ preferences, QoL, compliance and social aspects, all summarized in Figure 2 [19].

### 4.1. Multidisciplinary Decision Making for Therapeutic Options in the Relapsed/Refractory Setting

Within the last decades, many new drugs and various combinations have been approved for RRMM, and the decision that which therapy might be best for each individual patient has become more complex. Regarding this complexity, interdisciplinary conferences such as tumor boards are an important tool to ensure state-of-the-art antimyeloma treatment and to discuss individualized therapeutic approaches for symptomatic patients involving experts such as radiation therapists, orthopedists, radiologists, nephrologists, pathologists and specialists in molecular diagnostics [6,45].

### 4.2. First Relapse

Patients at first myeloma relapse should be assessed for eligibility for ASCT or novel agent treatment alone (Figure 3). Salvage-ASCT can be a valid treatment option in patients who remain transplant-eligible, i.e., if the remission after first ASCT lasted >18 months (resp. > 36 months with lenalidomide (R)-maintenance), at best even >2–5 years. According to retrospective studies, reinduction has shown no survival benefit in salvage-ASCT [31,65,67,68,69,70]. Young and fit patients with high-risk MM or early relapse ≤18 months (resp. ≤ 36 months with R-maintenance) after prior ASCT, may be considered for allogeneic stem-cell transplantation, ideally within a clinical trial [70].

For fit, but not transplant-eligible patients, a triplet combination should be administered including PIs and/or IMiDs and/or mAbs. In general, treatment regimens are preferably switched, rather than repeated. Following a bortezomib- and dexamethasone (Vd)-based first line therapy (without R or daratumumab (Dara)), Rd-based regimens, such as Dara-Rd/carfilzomib (K)-Rd/elotuzumab (Elo)-Rd/ixazomib (I)-Rd/RCd (Rd with cyclophosphamide) are recommended [19,30,65]. In R-refractory patients, Vd-, Kd- or pomalidomide and dexamethasone (Pd)-based regimens, including Dara-Vd/Dara-Kd/isatuximab (Isa)-Kd/VCd (bortezomib, cyclophosphamide, dexamethasone)/KCd (carfilzomib, cyclophosphamide, dexamethasone)/panobinostat (Pan)-Vd/PVd (pomalidomide, bortezomib, dexamethasone) or venetoclax (Ven)-Vd for t(11;14)) can be used [30,65]. When choosing an appropriate regimen, the licensing status of individual agents as well as therapy combinations needs to be kept in mind.

For frail patients at first relapse, doublets can be well-tolerated alternatives such as Rd in R-naïve/sensitive MM, or alternatively Vd or Kd in R-refractory disease. Dosing of the administered drugs should be adjusted as suggested in Figure 4 [10,71].

### 4.3. Second/Subsequent Relapse

**Figure 5:**
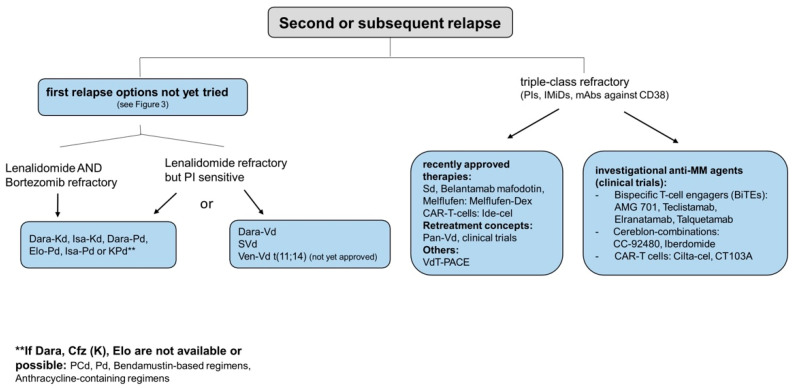
Treatment algorithm for RMM—2nd or subsequent relapse. After the establishment of a first and second generation of novel agents containing IMiDs, PIs and mAbs in antimyeloma therapy from 2004 to 2017, the past years have witnessed the development of more agents with diverse mechanisms of action that have proposed even more therapeutic options at second or subsequent relapse. Clinical trials at tertiary MM centers offer novel drug combinations with the aim of generating a more durable remission. Abbreviations: Belantamab-VRD = belantamab + bortezomib + lenalidomide + dexamethasone, BiTEs = bispecific T-cell engagers, CAR-T cells = chimeric antigen receptor T cells, CD-38 = cluster of differentiation 38, Cfz = carfilzomib, Cilta-cel = ciltacabtagene autoleucel, Dara = daratumumab, Dara-Kd = daratumumab + carfilzomib + dexamethasone, Dara-Pd = daratumumab + pomalidomide + dexamethasone, Dara-Vd = daratumumab + bortezomib + dexamethasone, Elo = elotuzumab, Elo-Pd = elotuzumab + pomalidomide + dexamethasone, Ide-cel = idecabtagen-vicleucel, IMiDs = immunomodulatory drugs, Isa-Kd = isatuximab + carfilzomib + dexamethasone, Isa-Pd = isatuximab + pomalidomide + dexamethasone, KPd = carfilzomib + pomalidomide + dexamethasone, mAb = monoclonal antibody, Melflufen-Dex = melflufen + dexamethasone, MM = multiple myeloma, Pan-V = panitumumab + bortezomib, Pcdpomalidomide + cyclophosphamide + dexamethasone, Pd = pomalidomide + dexamethasone, PI = proteasome inhibitor, RRMM = relapsed refractory multiple myeloma, Sd = selinexor + dexamethasone, SVd = selinexor + bortezomib + dexamethasone, VdT-PACE = bortezomib + dexamethasone + thalidomide + cisplatin + doxorubicin + cyclophosphamide + Etoposide, Ven-Vd = venetoclax + bortezomib + dexamethasone.

Preferably, any treatment option considered for first relapse, which the patient has not yet received, can be used in case of the second/subsequent relapse (Figure 5) [65,70].

The EHA/ESMO-guidelines suggest the use of Dara-Vd, selinexor (S)-Vd and Ven-Vd in R-refractory, but PI-sensitive patients. The BCL-2-inhibitor venetoclax has shown effectiveness in clinical trials in MM patients with translocation t(11;14), which is associated with higher BCL-2 expression in myeloma cells [72,73]. Once approved, venetoclax could be the first MM drug directly targeting a specific genetic mutation.

**Figure 6:**
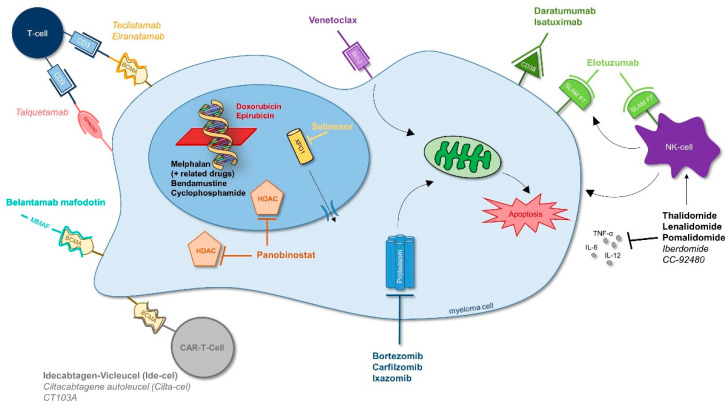
Scientific target sites for the treatment of MM. As modern research steadily unfolds new insights into the biology of MM and understanding mechanisms of resistance, scientific target sites for the treatment of MM diversify. Modern antimyeloma drugs currently tested or approved in the relapsed/refractory setting are encouraging especially in heavily pretreated MM patients. Abbreviations: Italic letters = not yet approved by EMA or FDA (dated June 2021), Bcl-2 = B-cell lymphoma 2, BCMA = B-cell maturation antigen, CD-38 = cluster of differentiation 38, GPRC5D = G protein-coupled receptor class C group 5 member D, HDAC = histone deacetylase, IL-6/-12 = interleukin 6/interleukin 12, MMAF = monomethyl auristatin F, NK-cell = natural killer cell, SLAM F7 = signaling lymphocytic activation molecule F7, TNF-α = tumor necrosis factor alpha, XPO1 = exportin 1.

For R- and V-refractory patients without prior antibody treatment, Dara-Kd, Isa-Kd, Dara-Pd, Elo-Pd and Isa-Pd are potential treatment options [70]. Especially for triple-class refractory patients, modern antimyeloma drugs with new modes of action need to be used (Figure 6).

Melflufen, FDA-licensed in combination with dexamethasone, is a lipophilic peptide-conjugated alkylating agent that targets aminopeptidases and leads to a rapid accumulation of melphalan metabolite in MM cells, showing activity in patients who were resistant to bortezomib and melphalan [74]. Selinexor inhibits the nuclear export protein XPO-1, which is overexpressed in myeloma cells and has shown efficacy in heavily pretreated RRMM patients, in combination with dexamethasone (Storm-), Vd (Boston-) or in various other combinations (Stomp-study) [75,76]. Belantamab-mafodotin is an antibody-drug conjugate targeting the B cell maturation antigen (BCMA), which is specific to plasma cells. It demonstrated single-agent activity in heavily pre-treated RRMM with a manageable safety profile and is now tested for longer responses in combination with pomalidomide, Vd, carfilzomib and various others [77]. Another propitious treatment approach is targeting BCMA with CAR-T-cells [78,79,80]. Idecabtagene vicleucel has been recently approved by the FDA as the first CAR-T-cell therapy against MM, and others are to follow shortly [71]. To exploit other options, retreatment concepts such as ‘backbone’-Panobinostat-Vd, licensed in patients with prior bortezomib treatment, and daratumumab showing potential in clinical trials, are of relevance in RRMM. Likewise, it is relevant to assess whether different CD38 antibodies, such as daratumumab and isatuximab, are beneficial to use after a CD38-treatment pause and in which sequence, after which pause (3–6 months) and with which additional MM-agents they are pursued in clinical trials. Other promising anti-MM treatment concepts, currently investigated in clinical trials, are shown in Figure 6. Among these are targeted approaches such as novel immunomodulatory agents (CELMoDs) and immunotherapies addressing various targets including BCMA, such as CAR-T cells and bispecific antibodies (teclistamab and elranatamab). Bispecific antibodies or T-cell engagers (BiTEs) link T-cells by binding CD3 and surface antigens of myeloma cells such as BCMA and result in T-cell activation and myeloma cell death. Encouraging targets include GPRC5D or FcRH5 [71,81,82,83,84,85]. The cereblon E3 ligase modulators CC-92480 and iberdomide are currently tested in pre-treated RRMM patients. Preclinical findings suggest that cereblon modulators can be effective in IMiD-refractory myeloma cells [86,87]. The role of checkpoint inhibitors such as pembrolizumab in the setting of RRMM has—at least with IMiDs—shown disappointing results, some of the reasons being summarized in a NEJM-perspective article [88], leading to their diminished use and non-approval in MM [88,89,90].

### 4.4. Supportives

In addition to the therapeutic options with direct effects against MM, supportives play a crucial role in symptomatic relief for RRMM patients. Since most myeloma patients suffer from osteolytic lesions, bisphosphonates or denosumab should be administered regularly [91]. Medical care of myeloma patients with skeletal lesions should include close collaboration with radiologists, orthopedists and radiation oncologists [6,45,91,92,93,94].

Therapeutic options also include symptom-orientated approaches with adequate pain medication and orthesis. In case of pathologic fractures or osteolysis with endangered skeletal stability, interdisciplinary discussion of surgical options and radiation therapy is essential.

Moreover, most myeloma patients suffer from an impaired immune response due to antimyeloma treatment and immunoparesis with resulting susceptibility to infections. Prophylactic medication to prevent pneumocystis, reactivation of herpes zoster and various other infectious risks, including essential vaccinations (e.g., for COVID-19) should be ensured [95,96].

Other supportive drugs may be necessary depending on the drug regimen used (e.g., thromboprophylaxis in IMiDs or anti-anaphylactic premedication for mAbs); for more information, the respective summary of product characteristics can be consulted. Substitution of intravenous polyvalent immunoglobulins may be considered in patients with frequently recurrent or severe infections due to a lack of functional antibodies [97,98].

## 5. Conclusions

To date, MM is considered an incurable disease, and almost all patients will experience one or more relapses. Nevertheless, over the last decades, novel insights into the biology of the disease have resulted in the development and approval of many novel agents and drug combinations, starting in RRMM and developing to newly diagnosed MM. Therefore, decisions for ideal antimyeloma treatment for individual patients have become more complex, and disease-, treatment- and patient-related factors should be considered. Since MM typically affects older patients, balancing treatment efficacy, tolerability, comorbidities and vulnerability are increasingly challenging for treating physicians. In this setting, interdisciplinary tumor boards play a crucial role in treatment decisions, supportives and multidisciplinary intervention needs. MM-specific comorbidity indices, such as the R-MCI or IMWG frailty index, and others, plus geriatric functional assessments are important tools for obtaining a more objective evaluation of patients’ real constitution and treatment endurance. Most importantly, these assessments should be easy to perform in clinical practice, clearly allowing to define fit vs. frail patients and thereby supporting individual treatment decisions in order to avoid over- and under-treatment. The future focus should be on more precise disease monitoring via MRD diagnostics, innovative-targeted therapies and immunotherapeutic options. The promotion of clinical trials for elderly and frail patients, including interdisciplinary incentives, should further enhance prospects, PFS, OS, therapy tolerance and QoL for these highly relevant, ever-increasing patients.
